# Predictors of long-term smoking cessation: results from the global adult tobacco survey in Poland (2009–2010)

**DOI:** 10.1186/1471-2458-12-1020

**Published:** 2012-11-22

**Authors:** Dorota Kaleta, Przemysław Korytkowski, Teresa Makowiec-Dąbrowska, Bukola Usidame, Leokadia Bąk-Romaniszyn, Adam Fronczak

**Affiliations:** 1 Department of Preventive Medicine, Medical University of Łódź, Łódź, Poland; 2 Faculty of Computer Science and Information Technology, West Pomeranian University of Technology in Szczecin, Szczecin, Poland; 3 Department of Work Physiology and Ergonomics, Nofer Institute of Occupational Medicine, Łódź, Poland; 4 Public Health Faculty, Medical University of Łódź, Łódź, Poland; 5 Department of Public Policy, University of Massachusetts, Boston, USA; 6 Department of Nutrition in Digestive Tract Diseases, Medical University of Łódź, Łódź, Poland; 7 Department of Biopharmacy, Medical University of Łódź, Łódź, Poland

**Keywords:** Tobacco smoking cessation, Socio-demographic factors, Adults, GATS, Poland

## Abstract

**Background:**

Expanding the information on determinants of smoking cessation is crucial for developing and implementing more effective tobacco control measures at the national as well as European levels. Data on smoking cessation and its social correlates among adults from middle-income countries of Central and Eastern Europe are still poorly reported in the literature. The aim of the study was to analyze the association of socio-demographic indicators with long term tobacco smoking cessation (quit smoking for at least one year prior to interview) among adults. Moreover, we evaluated motives for giving up smoking from former smokers.

**Methods:**

Data on former as well as current smokers’ socio-demographic and smoking-related characteristics were derived from the Global Adult Tobacco Survey (GATS). GATS is a cross-sectional, nationally representative household survey implemented in Poland between 2009 and 2010. GATS collected data on a representative sample of 7,840 individuals including 1,206 individuals who met the criteria of long-term smoking cessation and 2,233 current smokers. Smoking cessation rate was calculated as the number of former smokers divided by the number of ever smokers. Logistic regression analyses were used to obtain odds ratios (ORs) and 95% confidence interval (CI) of the broad number of variables on successful cessation of smoking.

**Results:**

Among females the quit rate was 30.4% compared to 37.9% in males (p < 0.01). Former smokers declared concerns about the health hazard of smoking (60.8%) and the high price of cigarettes (11.6%) as primary reasons for smoking cessation. Older age, high education attainment, awareness of smoking health consequences was associated with long-term quitting among both genders. Also employed males had over twice the probability of giving up smoking compared with unemployed, and being religious did not contribute to successful smoking cessation.

**Conclusion:**

Results indicated that smoking cessation policies focused on younger age groups are vital for curbing tobacco epidemic in Poland and should become a public health main concern. There is also the need for interventions to raise awareness on smoking health risks and quitting benefits are crucial to increase cessation potential among adult smokers. Nevertheless further effort needs to be done to prevent smoking uptake.

## Background

The most recent report launched by the World Health Organization in 2012 indicated that the burden of deaths attributable to tobacco use is still very high in Europe [1]. The death rate from non-communicable diseases (NCDs-1527 per 100,000 population) was about 21.9 times that for communicable diseases (70 per 100,000 population). Tobacco was responsible for 18% of all NCDs. Within the NCDs category, ischemic heart disease accounted for 437 deaths per 100,000 population aged 30 years and above, with 16% these deaths attributed to tobacco. Cancer of the trachea, bronchus and lungs accounted for 71 deaths per 100,000 population with 85% of these deaths attributable to tobacco. The death rate due to tobacco related diseases in men was 467 per 100,000 men at age 30 years and more, compared with women, 117 per 100,000 women at age 30 years and more. The proportion of deaths attributable to tobacco was close to 25% in men and 7% in women. Unfortunately, in Poland these indicators are even higher and the data is alarming [1].

In Poland, tobacco was responsible for 23% of all NCDs, 90% of trachea, bronchus and lung cancer. The proportion of deaths attributable to tobacco was close to 31% in men and 12% among women aged 30 years and more [1]. The most important aspect is that smoking related deaths are potentially avoidable. Smoking cessation has significant health benefits for smokers at all ages [2]. Increasing the cessation rate is considered the only strategy that can determine a significant reduction in smoking-related mortality among adults in the short term [3]. Interventions that can increase the smoking cessation rate on the population level could save many lives [4].

During the past 30 years, high numbers of smokers in the developed countries have given up tobacco smoking [5]. According to the World Health Organization data, in the 1980s the smoking prevalence in Poland was the highest in the country’s history and one of the highest in Europe, including Central and Eastern Europe [6]. In 1982, the percentage of smokers among young and middle-aged men was 70%, and 50% in a similar-age group of women. Among men, the fall in ratio of daily smokers occurred in all age groups from around 60% in 1982 to 40% in 2000–2004, reaching approximately 1% rate of annual decline among all men [6]. Among women, the highest decline in incidence of daily smoking occurred in the youngest age group (20–29 years) from 50% to 25%. Unfortunately, there is evidence in recent years of a slowdown in the rate of decline in smoking prevalence among men, and of a halt in the falling trend among young adult women [6]. Therefore, there is a need to increase the effectiveness of tobacco control measures. Obtaining information on predictors of successful cessation can contribute significantly to improving tobacco control activities on an individual and population level.

Studies that have investigated determinants of tobacco quitting are not homogeneous in terms of methodological approach [7,8]. They range in design from cross-sectional, follow-up studies to intervention trials [3,9-12]. Many of these approaches are limited to constricted subgroups [13]. Often surveys have been conducted in populations at high risk of heart disease or cancer patients, and they have a limited range of variation on such variables as age, race, and socioeconomic status [14-20].

Still, little attention is paid to the demographic and social correlates of successful smoking cessation among adult Poles in our country. Evidently more research is needed to update evidence-based data to develop well-targeted tobacco control activities.

The aim of this study was to analyze the association of socio-demographic indicators with the long term tobacco smoking cessation among adults in Poland. Moreover, we assessed the motivations for quitting smoking cigarettes among former smokers.

## Methods

Data on former and current smokers’ socio-demographics, as well as smoking-related characteristics, and reasons for smoking cessation were derived from the Global Adult Tobacco Survey (GATS) which was described in detail elsewhere [21]. Global Adult Tobacco Survey is a cross-sectional, nationally representative household survey [22]. The target population was non-institutional residents aged 15 years and older in all 16 voivodeships of Poland [23]. The GATS Poland sample was selected in three stages, where statistical regions were treated as Primary Sampling Units (PSUs). In the first stage of sample selection a total of 200 urban PSUs and 200 rural PSUs were selected with probability proportionate to size according the GATS sample selection requirements.

In the second stage of sample selection 36 households were selected from each urban PSU and 34 households were selected from each rural PSU using simple random sampling without replacement from the National Official Territorial Division Register (TERYT). One individual (eligible man or woman) from each of the participating households were randomly selected. A total of 6800 households were selected (3600 male and 3200 female) from rural PSUs with 7200 households (3800 male and 3400 female) drawn from urban PSUs resulting into a total sample of 14000 non-institutionalized households from all 16 voivodeships for GATS Poland. GATS collected data from 7,840 sampled individuals. GATS data were collected by trained pollsters during the face to face interviews between 2009 and 2010.

Data used for the study is publicly available from the website of the Global Tobacco Surveillance System (GTSS). GTSS is a Web-based data application that houses and displays data from four tobacco-related surveys conducted around the world. The purpose of GTSS is to enhance countries’ capacity to monitor tobacco use, guide national tobacco prevention, and control programs, and facilitate comparison of tobacco-related data at the national, regional, and global levels.

### Questionnaire

The GATS questionnaire covered socio-demographic issues, detailed demands on current smoking and smoking history [24]. It includes questions on the date smokers began cigarette smoking on a daily basis or the number of years since quitting for ex-smokers. Moreover, the questionnaire determined if the respondent went to a doctor or health care provider in the past 12 months, and whether a respondent was advised by a doctor or health care provider (during any visit in the past 12 months) to quit smoking tobacco. GATS allows to determine whether those who attempted to quit used any of the following aid “counseling by a specialist even at a smoking cessation clinic; used nicotine replacement therapy, e.g. chewing gum, patches, tablets, inhaler and other agents containing nicotine or other prescription drugs, e.g. Tabex, Zyban, Champix; other pharmaceutical agents, quit line advice/helpline, or switching to smokeless tobacco” during the last 12 months. Furthermore GATS questionnaire provided information on the primary reason for quitting smoking cigarettes of respondents [24].

### Study variables

The category for ever smokers covered current and ex-smokers subgroups. A current smoker was defined as someone who had smoked more than an average of one cigarette per day on a regular basis for at least one year. A former smoker was defined as someone who had given up smoking for at least one year prior to the interview, capturing our definition of successful smoking cessation [25]. Those respondents who had given up smoking more recently were considered current smokers. Following the analysis previously performed by Kabat et al. overall stopping rates or “quit rates” [rates between ex- and ever-smokers] were calculated for smokers with different characteristics, as the number of former smokers divided by the number of ever smokers and multiplied by 100% [7]. Data on gender and age of the respondents were also included in our analysis in addition to information on age at smoking onset. Age at smoking onset was characterized as the age at which respondents started to smoke tobacco on a regular basis (≤17, 18–20, 21 years or over). We also used in our analysis the data on educational attainment of all subjects. Educational level was regarded as: primary education, vocational education, secondary education, and higher education.

The measure of economic activity classified subjects currently with permanent job as employed, currently with no permanent job as unemployed, and pupils, students, persons occupied with household keeping, retired, pensioners due to disability as economically non-active. Furthermore, respondents were asked whether their place of residence was a rural or urban area (urban area up to 50 000, from 50 000 to 200 000, and over 200 000 inhabitants). We also took into consideration the awareness of the negative health consequences of smoking. We categorized our respondents as aware (those who answered “yes” to the question: Do you think that tobacco smoking causes serious diseases?) and not aware (those who answered “no” and “do not know”). We also split our subjects according to being religious or not. Moreover, people who declared to be religious were categorized as a: believer practicing regularly; believer but not practicing regularly; believer but not practicing. Reasons stated for quitting smoking cigarettes by respondents were recorded in five categories: cigarettes became too expensive for them, they realized that smoking harms, someone they know decided to quit, now there are less public places where they can smoke, or some other reason.

### Statistical analyses

Statistical associations of the given categories of characteristics in the analyzed groups were assessed with the chi-square test. All analyses were performed separately for men and women in six age groups: under 25, 25–29, 30–39, 40–49, 50–59, 60 years and older. For the comparison of proportions, a chi-square test was used. We used univariate and multivariate logistic regression analyses of unweighted data to obtain odds ratios (ORs) and 95% confidence interval (CI) of each indicator on smoking cessation. In the first stage crude coefficients – odds ratios (OR) of the impact of odd variables on the successful smoking cessation in males and females were calculated. This was followed by a multifactorial analysis considering the simultaneous effect of all variables on the possibility of successful smoking cessation. All p values were two-sided and p < 0.05 was used to denote statistical significance. The STATISTICA Windows XP version 8.0 program was used to perform the statistical analysis.

## Results

Among the 14 000 households selected for the survey, 8948 (63.9%) households and 7840 (93.9%) sampled persons successfully completed the interviews. The overall survey participation rate was 65.1%. Of the 7840 respondents, 1206 subjects (811 men and 395 women) had quit smoking and did not smoke for at least 1 year before the interview. Current smokers were 1330 male and 903 female. Distribution of former, current, and ever smokers and quit rates of the study sample by gender and selected characteristics are available in Table 1.

**Table 1:** Characteristics of former, current, ever smokers and quit rates by gender – Global Adult Tobacco Survey Poland 2009–2010

**Characteristic**	**Male N = 2141**				**Female N = 1298**			
	**Former smokers N = 811**	**Current smokers N = 1330**	**Ever smokers N = 2141**	**Quit rate# %**	**Former smokers N = 395**	**Current smokers N = 903**	**Ever smokers N = 1298**	**Quit rate# %**
	**n (%)**	**n (%)**	**n (%)**		**n (%)**	**n (%)**	**n (%)**	
**Age (years)**								
<25	13 (1.6)	117 (8.8)	130 (6.1)	10.0	11 (2.8)	72 (8.0)	83 (6.4)	13.3
25–29	32 (3.9)	147 (11.1)	179 (8.4)	17.9	31 (7.8)	96 (10.6)	127 (9.8)	24.4
30–39	93 (11.5)	307 (23.1)	400 (18.7)	23.3	75 (19.0)	171 (18.9)	246 (19.0)	30.5
40–49	137 (16.9)	303 (22.8)	440 (20.6)	31.1	62 (15.7)	220 (24.4)	282 (21.7)	22.0
50–59	168 (20.7)	282 (21.2)	450 (21.0)	37.3	98 (24.8)	246 (27.2)	344 (26.5)	28.5
≥60	368 (45.4)	174 (13.1)	542 (25.3)	67.9	118 (29.9)	98 (10.9)	216 (16.6)	54.6
**Age at smoking onset**								
≤17	315 (38.8)	548 (41.2)	863 (40.3)	36.5	95 (24.1)	262 (29.0)	357 (27.5)	26.6
18–20	362 (44.6)	551 (41.4)	913 (42.6)	39.6	194 (49.1)	400 (44.3)	594 (45.8)	32.7
≥21	134 (16.5)	231 (17.4)	365 (17.0)	36.7	106 (26.8)	241 (26.7)	347 (26.7)	30.5
**Education**								
primary	202 (24.9)	203 (15.3)	405 (18.9)	49.9	55 (13.9)	112 (12.4)	167 (12.9)	32.9
vocational	264 (32.6)	584 (43.9)	848 (39.6)	31.1	100 (25.3)	257 (28.5)	357 (27.5)	28.0
secondary	257 (31.7)	428 (32.2)	685 (32.0)	37.5	163 (41.3)	399 (44.2)	562 (43.3)	29.0
high	88 (10.9)	115 (8.6)	203 (9.5)	43.3	77 (19.5)	135 (15.0)	212 (16.3)	36.3
**Occupational classification**								
non-economically active	425 (52.4)	327 (24.6)	752 (35.1)	56.5	217 (54.9)	355 (39.3)	572 (44.1)	37.9
employed	355 (43.8)	864 (65.0)	1219 (56.9)	29.1	155 (39.2)	488 (54.0)	643 (49.5)	24.1
unemployed. currently with no permanent job	27 (3.3)	137 (10.3)	164 (7.7)	16.5	22 (5.6)	59 (6.5)	81 (6.2)	27.2
**Place of residence**								
rural	456 (56.2)	690 (51.9)	1146 (53.5)	39.8	169 (42.8)	364 (40.3)	533 (41.1)	31.7
**urban**								
up to 50 000	151 (18.6)	234 (17.6)	385 (18.0)	39.2	83 (21.0)	158 (17.5)	241 (18.6)	34.4
50 000–200 000	80 (9.9)	176 (13.2)	256 (12.0)	31.3	63 (15.9)	163 (18.1)	226 (17.4)	27.9
over 200 000	124 (15.3)	230 (17.3)	354 (16.5)	35.0	80 (20.3)	218 (24.1)	298 (23.0)	26.8
**Religiosity**								
believer practicing regularly	441 (54.4)	483 (36.3)	924 (43.2)	47.7	198 (50.1)	327 (36.2)	525 (40.4)	37.7
believer but practicing not regularly	220 (27.1)	481 (36.2)	701 (32.7)	31.4	127 (32.2)	364 (40.3)	491 (37.8)	25.9
believer but not practicing	98 (12.1)	287 (21.6)	385 (18.0)	25.5	50 (12.7)	160 (17.7)	210 (16.2)	23.8
not religious	28 (3.5)	53 (4.0)	81 (3.8)	34.6	8 (2.0)	28 (3.1)	36 (2.8)	22.2
**Awareness of smoking health consequences**								
yes	774 (95.4)	1081 (81.3)	1855 (86.6)	41.7	374 (94.7)	742 (82.2)	1116 (86.0)	33.5
no	14 (1.7)	160 (12.0)	174 (8.1)	8.0	8 (2.0)	78 (8.6)	86 (6.6)	9.3

#Quit rates = no. former smokers/no. ever smokers x100.

The mean age of ever-smokers was 48.0 ± 16.0 years in men compared to 45.8 ± 14.1 years in women (p < 0.01). In the group of current male smokers mean age was 43.0 ± 14.0 vs. 43.8 ± 13.1 years in female, respectively (p > 0.05). The mean age of former smokers was 56.2 ± 15.6 years in males and 50.3 ± 15.5 years in females (p < 0.001).

Women started smoking at a later age than men (data not presented in the tables). While the mean age when smoking began was 18.4 ± 3.7 and 18.3 ± 3.6 years in former and current male smokers respectively, it was 20.0 ± 4.4 (men vs. women p < 0.001) and 20.0 ± 4.6 years in the former and current female smokers respectively (men vs. women p < 0.001). Among females the quit rate was 30.4% compared to 37.9% in males (p < 0.01). In addition, women quit at a younger age than men. The mean age of quitting for male and female former smokers was 41.5 ± 13.5 and 38.1 ± 13.3 years respectively (p < 0.001). Men had been smoking 23.1 ± 13.3 years before quitting. Duration of smoking habit was 18.2 ± 12.2 years in the group of female former smokers (males vs. females p < 0.001). Male former smokers reported 14.7 ± 11.8 and females 12.1 ± 10.0 years since quitting (p < 0.001). From the group of 1206 former smokers (including 811 men and 395 women) 24 respondents (16 men and 8 women) chose “don’t know” answer to the question on reasons for quitting smoking and thus were not considered in further calculations. In the group of male former smokers, primary reasons for quitting smoking were: they realized that smoking harms (n = 533, 67%), cigarettes became too expensive for them (n = 100, 12.6%), someone they know decided to quit (n = 32, 4.1%), now there are less public places where they can smoke (n = 1, n = 0.1%), or some other reason (n = 129, 16.2%). Also in the group of female former smokers, primary reasons for quitting smoking were: they realized that smoking harms (n = 200, 51.7%), cigarettes became too expensive for them (n = 40, 10.3%), someone they know decided to quit (n = 25, 6.5%), or some other reason (n = 121, 31.3%). The detailed distribution of reasons for quitting tobacco smoking among former smokers according to selected socio-demographics is presented in Table 2.

**Table 2:** Reasons to quit among former smokers by gender and selected characteristics – Global Adult Tobacco Survey Poland 2009–2010

**Characteristic**	**Male N = 795**					**Female N = 387**				
	**cigarettes became too expensive N = 100**	**realized smoking is harmful N = 533**	**someone decided to quit N = 32**	**less public places N = 1**	**Other N = 129**	**cigarettes became too expensive N = 40**	**realized smoking is harmful N = 200**	**someone decided to quit N = 25**	**less public places N = 1**	**Other N = 121**
	**n (%)**	**n (%)**	**n (%)**	**n (%)**	**n (%)**	**n (%)**	**n (%)**	**n (%)**	**n (%)**	**n (%)**
**Age (years)**										
<25	3 (23.1)	8 (61.5)	0 (0.0)	0 (0.0)	2 (15.4)	3 (27.3)	4 (36.4)	0 (0.0)	0 (0.0)	4 (36.4)
25–29	6 (19.4)	17 (54.8)	0 (0.0)	0 (0.0)	8 (25.8)	4 (12.9)	14 (45.2)	1 (3.2)	0 (0.0)	12 (38.7)
30–39	13 (14.3)	59 (64.8)	5 (5.5)	0 (0.0)	14 (15.4)	3 (4.2)	40 (55.6)	3 (4.2)	0 (0.0)	26 (36.1)
40–49	22 (16.5)	91 (68.4)	2 (1.5)	0 (0.0)	18 (13.5)	5 (8.1)	33 (53.2)	9 (14.5)	1 (1.6)	14 (22.6)
50–59	21 (12.7)	109 (66.1)	9 (5.5)	0 (0.0)	26 (15.8)	13 (13.4)	45 (46.4)	5 (5.2)	0 (0.0)	34 (35.1)
≥60	35 (9.7)^a^	249 (68.8)	16 (4.4)	1 (0.3)	61 (16.9)	12 (10.5)	64 (56.1)	7 (6.1)	0 (0.0)	31 (27.2)
**Education**										
primary	24 (12.2)	132 (67.3)	11 (5.6)	0 (0.0)	29 (14.8)	6 (11.3)	29 (54.7)	2 (3.8)	0 (0.0)	16 (30.2)
vocational	39 (14.9)	180 (68.7)	10 (3.8)	0 (0.0)	33 (12.6)	15 (15.6)	50 (52.1)	5 (5.2)	0 (0.0)	26 (27.1)
secondary	33 (13.1)	168 (66.9)	5 (2.0)^c^	0 (0.0)	45 (17.9)	16 (9.9)	77 (47.8)	12 (7.5)	0 (0.0)	56 (34.8)
high	4 (4.7) ^b^	53 (61.6)	6 (7.0)^d^	1 (1.2)	22 (25.6) ^e,f^	3 (3.9)^g^	44 (57.1)	6 (7.8)	1 (1.3)	23 (29.9)
**Occupational classification**										
non- economically active	42 (9.8)	199 (46.5)	134 (31.3)	0 (0.0)	53 (12.4)	14 (9.8)	76 (53.1)	12 (8.4)	1 (0.7)	40 (28.0)
employed	49 (14.2)	226 (65.3)^j^	14 (4.0)^l^	0 (0.0)	57 (16.5)	17 (11.1)	80 (52.3)	12 (7.8)	1 (0.7)	43 (28.1)
unemployed	8 (29.6) ^h,i^	10 (37.0)^k^	0 (0.0)^m^	0 (0.0)	9 (33.3) ^n,o^	2 (9.5)	10 (47.6)	1 (4.8)	0 (0.0)	8 (38.1)
**Place of residence**										
rural	60 (13.5)	300 (67.4)	20 (4.5)	0 (0.0)	65 (14.6)	21 (12.8)	85 (51.8)	10 (6.1)	0 (0.0)	48 (29.3)
**urban**										
up to 50 000	18 (12.2)	99 (67.3)	4 (2.7)	1 (0.7)	25 (17.0)	8 (9.6)	35 (42.2)	6 (7.2)	1 (1.2)	33 (39.8)
50 000– 200 000	10 (12.5)	49 (61.3)	4 (5.0)	0 (0.0)	17 (21.3)^p^	7 (11.7)	32 (53.3)	2 (3.3)	0 (0.0)	19 (31.7)
over 200 000	12 (9.8)	85 (69.1)	4 (3.3)	0 (0.0)	22 (17.9)	4 (5.0)	48 (60.0)^r^	7 (8.8)	0 (0.0)	21 (26.3)

^a^ p < 0,04 age ≥60 vs age 40–49; ^b^ p < 0,03 high education vs vocational; ^c^ p < 0,05 secondary education vs primary; ^d^ p < 0,03 high education vs secondary; ^e^ p < 0,04 high education vs primary.

^f^ p < 0,005 high education vs vocational; ^g^ p < 0,03 high education vs vocational; ^h^ p < 0,04 unemployed currently with no permanent job vs employed; ^i^ p < 0,02 unemployed currently with no permanent job vs non-economically active; ^j^ p < 0,001 employed vs non-economically active; ^k^ p < 0,004 unemployed currently with no permanent job vs employed; ^l^ p < 0,001 employed vs non-economically active ^m^ p < 0,001 unemployed currently with no permanent job vs non-economically active; ^n^ p < 0,003 unemployed currently with no permanent job vs non-economically active.

^o^ p < 0,03 unemployed currently with no permanent job vs employed; ^p^ p < 0,04 place of residence urban 50thous.-200 thous vs rural; ^r^ p < 0,03 place of residence urban over 200 thous vs urban up to 50 thous.

During 12 months prior to the interview 27.0% (n = 359) of male current smokers and 29.7% (n = 268) of female current smokers (p > 0.05) attempted to give up smoking. In this group only 7.7% of men and 11.4% of women (p < 0.04) used professional aid in trying to stop smoking tobacco. In most cases, respondents used pharmacotherapy. Nicotine replacement therapy was the most often used (4.5% males, 6.6% females; p < 0.004), and in second place drugs like Tabex or Zyban (1.2% males vs. 2.2% females; p > 0.05). Almost half of the current male smokers - 47.1% (n = 528) and 39.2% (n = 297) of the current female smokers had no plans to quit (p < 0.001). Other respondents considered giving up smoking in the future. In the 12 months preceding the survey 1331 (including 173 men and 618 women; p < 0.001) current smokers saw a doctor or other health care provider from various reasons. Out of this group, the expert discussed tobacco smoking with 61.5% men and 59.0% women (p > 0.05), while 45.9% men and 43.5% women were advised to quit smoking (p > 0.05).

### Univariate analysis

The results of the univariate and multivariate regression analyses are presented in Tables 3 and 4. In men, the long-term quit rates increased with age. The quit rates were highest among the male subjects of 60 years of age or older compared to those aged 25–29 years (OR = 19.0; 95% CI: 10.4–34.7; p < 0.0001). Women who were age 60 years or older had an increased like hood of long-term smoking cessation (p < 0.0001). Successful cessation was associated with education. Male subjects who declared vocational education had lower probabilities of long-term smoking cessation compared with the highly educated ones (men OR = 0.6; 95% CI: 0.4–0.8; p < 0.001, women OR = 0.7; 95% CI: 0.5–1.0; p < 0.05). Female subjects who had vocational education achieved also a lower quit rate compared to those with higher education (OR = 0.7; 95% CI: 0.5–1.0; p < 0.05). Among the male population, the likelihood of quitting successfully was more than 6.5 times higher in the category of non-economically active compared to the unemployed group (OR = 6.6; 95% CI: 4.3–10.21). Also employed males had over twice the probability of giving up smoking compared with unemployed (OR = 2.1; 95% CI: 1.3–3.2). Employment status was not significantly associated with long-term quitting in women. The residents of urban areas of 50 000 to 200 000 of inhabitants were less successful in quitting compared to the people living in rural settings (p < 0.05). In men, those who considered themselves religious and practiced regularly were significantly more likely to quit smoking for one year or longer compared to the non-religious ones (OR = 1.7; 95% CI: 1.1–2.8; p < 0.05). On the other hand, place of residence and being a religious person were not significantly associated with long-term quitting in women. Among the male population, the rate of ex-smokers was over eight times higher in the group that considered smoking to cause serious illnesses, compared to the people that did not perceive smoking as dangerous to health (OR = 8.2; 95% CI: 4.7–14.2; p < 0.0001). Among women awareness of smoking health consequences contributed to quitting successfully as well (OR = 4.9; 95% CI: 2.3–10.3; p < 0.0001). In the univariate analysis, age at smoking onset was not significantly associated with long-term quitting among men. In women age at smoking onset was significantly associated with long-term quitting (OR = 1.7; 95% CI: 1.1–2.8; p < 0.05).

**Table 3:** Odds Ratios (OR) and 95% Confidence Intervals (CI) for long-term smoking cessation to selected socio-demographic characteristics in men – Global Adult Tobacco Survey Poland (2009–2010)

**Variable**	**Total (n)**	**Quit rate**	**Univariate logistic regression**		**Multivariate logistic regression**^**a**^	
			**OR**	**95% CI**	**OR**	**95% CI**
**Age (years)**						
<25	130	10.0	1.00	reference	1.00	reference
25–29	179	17.9	1.96	0.98– 3.90	1.94	0.95– 3.94
30–39	400	23.3	2.73 c	1.47 – 5.06	3.05 d	1.60–5.80
40–49	440	31.1	4.07 d	2.22– 7.47	4.99 d	2.65–9.41
50–59	450	37.3	5.36 d	2.93 – 9.81	6.42 d	3.44–11.99
≥60	542	67.9	19.03 d	10.44– 34.71	19.47 d	10.13– 37.43
**Age at smoking onset**						
≤17	863	26.6	1.00	reference	1.00	reference
18–20	913	32.7	1.14	0.94–1.38	0.88	0.71–1.11
≥21	365	30.5	1.01	0.78–1.30	0.58	0.42–0.76
**Education**						
primary	405	49.9	1.30	0.93–1.82	0.67	0.42–1.06
vocational	848	31.1	0.59 c	0.43 –0.81	0.59 b	0.39 –0.88
secondary	685	37.5	0.78	0.57–1.08	0.93	0.62–1.39
high	203	43.3	1.00	reference	1.00	reference
**Occupational classification**						
economically not active	752	56.5	6.60 d	4.26–10.21	2.10 c	1.29–3.41
employed	1219	29.1	2.08 d	1.35–3.21	1.74 b	1.11–2.71
unemployed	164	16.5	1.00	reference	1.00	reference
**Place of residence**						
rural	1146	39.8	1.00	reference	1.00	reference
**urban**						
up to 50 000	385	39.2	0.99	0.77–1.24	1.07	0.81–1.40
50 000–200 000	256	31.3	0.69 b	0.51–0.92	0.71	0.50–1.00
over 200 000	354	35.0	0.82	0.63–1.04	0.97	0.72–1.32
**Religiosity**						
believer practicing regularly	924	47.7	1.73 b	1.07–2.78	1.05	0.66 –1.66
believer but practicing not regularly	701	31.4	0.87	0.53–1.41	0.73	0.46–1.16
believer but not practicing	385	25.5	0.65	0.39–1.08	0.58 b	0.36–0.94
not religious	81	34.6	1.00	reference	1.00	reference
**Awareness of smoking health consequences**						
yes	1855	41.7	8.18 d	4.70–14.24	5.58 d	3.80–8.15
no	174	8.0	1.00	reference	1.00	reference

^a^ Fully adjusted model including: age, age at smoking onset, Education,. occupational classification, place of residence, Religiosity, awareness of smoking health consequences.

^b^ p ≤ 0.05.

^c^ p ≤ 0.01.

^d^ p ≤ 0.001.

**Table 4:** Odds Ratios (OR) and 95% Confidence Intervals (CI) for long-term smoking cessation to selected socio-demographic characteristics in women – Global Adult Tobacco Survey Poland (2009–2010)

**Variable**	**Total (n)**	**Quit rate**	**Univariate logistic regression**		**Multivariate logistic regression**^**a**^	
			**OR**	**95% CI**	**OR**	**95 CI**
**Age (years)**						
<25	83	13.3	1.00	reference	1.00	reference
25–29	127	24.4	2.11	0.99–4.47	2.15	0.99–4.66
30–39	246	30.5	2.87 c	1.44– 5.72	3.34 c	1.63–6.87
40–49	282	22.0	1.84	0.92– 3.69	2.27 b	1.09–4.72
50–59	344	28.5	2.61 c	1.33– 5.13	3.15 c	1.55–6.40
≥60	216	54.6	7.88 d	3.96–15.69	8.42 d	3.99–17.80
**Age at smoking onset**						
≤17	357	26.6	1.00	reference	1.00	reference
18–20	594	32.7	1.34 b	1.00–1.79	1.04	0.76–1.43
≥21	347	30.5	1.21	0.88–1.68	0.74	0.51–1.07
**Education**						
primary	167	32.9	0.86	0.56–1.32	0.52 b	0.30–0.92
vocational	357	28.0	0.68 b	0.47–0.98	0.55 b	0.34–0.88
secondary	562	29.0	0.72	0.51–1.00	0.64 b	0.42 –0.97
high	212	36.3	1.00	reference	1.00	reference
**Occupational classification**						
economically not active	572	37.9	1.64	0.98–2.75	1.18	0.68 –2.05
employed	643	24.1	0.85	0.50–1.43	0.79	0.46–1.36
unemployed	81	27.2	1.00	reference	1.00	reference
**Place of residence**						
rural	533	31.7	1.00	reference	1.00	reference
**urban**						
up to 50 000	241	34.4	1.13	0.82–1.56	1.11	0.78–1.58
50 000–200 000	226	27.9	0.83	0.59–1.17	0.81	0.56–1.19
over 200 000	298	26.8	0.79	0.58–1.08	0.81	0.57–1.16
**Religiosity**						
believer practicing regularly	525	37.7	2.12	0.95– 4.74	1.66	0.91– 3.03
believer but practicing not regularly	491	25.9	1.22	0.54–2.75	1.12	0.62 –2.05
believer but not practicing	210	23.8	1.09	0.47– 2.55	1.03	0.53–1.98
not religious	36	22.2	1.00	reference	1.00	Reference
**Awareness of smoking health consequences**						
yes	1116	33.5	4.91 d	2.35–10.28	4.39 d	2.69–7.18
no	86	9.3	1.00	reference	1.00	reference

^a^ Fully adjusted model including: age, age at smoking onset, education, occupational classification, place of residence, Religiosity, awareness of smoking health consequences.

^b^ p ≤ 0.05.

^c^ p ≤ 0.01.

^d^ p ≤ 0.001.

### Multivariate analysis

Age occurred as a significant, independent predictor of the long-term tobacco quitting among men and women (Tables 3 and 4). The odds of smoking cessation increased with age and were highest for the oldest age group (p < 0.0001). Moreover, low education attainment was negatively associated with quitting smoking for both genders. The quit rate was lower among men who reported vocational education than among men with higher education (OR = 0.6; 95% CI 0.4–0.9; p < 0.05). Quitting probability was decreased among women who reported lower educational levels (primary/vocational/secondary) than among women with higher education (primary: OR = 0.5; 95% CI 0.3–0.9; vocational: OR = 0.5; 95% CI 0.3–0.9; secondary: OR = 0.6; 95% CI 0.4–1.0; p < 0.05). Employment status was not associated with cessation likelihood among women, while this association was observed among men. The probability of long-term smoking cessation was over 2 times higher among respondents who were not economically active (OR = 2.1; 95% CI: 1.29–3.4; p < 0.001), and about 2 times higher for employed men (OR = 1.7; 95% CI: 1.1–2.7; p < 0.05) compared with the unemployed subjects. Significantly higher probability of cessation was among men (OR = 5.6; 95% CI: 3.8–8.1; p < 0.0001) and women (OR = 4.4; 95% CI: 2.7–7.1; p < 0.0001) who were aware of negative health consequences compared to those not aware., Being religious but not practicing was negatively correlated with successful smoking cessation among males (p < 0.05). Religiosity was not associated with cessation among females. Age of onset smoking and place of residence did not influence smoking rates in both genders.

## Discussion

This study demonstrates the association of long term cessation with socio-demographic predictors among a representative sample of Poles. This association remained after adjustment of other important variables. Several previous studies conducted with other populations have demonstrated similar associations [3,7,9,12]. Direct comparisons of GATS results with other studies are challenging due to diverse study designs, country profiles, different tobacco control policies among countries and time of conducting studies. Nonetheless, GATS identified that older age strongly predicted cessation, which is consistent with other studies [10,26,27]. The most common interpretation of this association is that with increasing age of subjects, the health worsens and increases the prevalence of symptoms caused by tobacco related diseases [7,28]. The study of Sieminska et al. showed that poor health motivates people to quit in order to reduce negative health effects of tobacco use, and many smokers are not successful in quitting before the manifestation of a severe disease [16]. Other researchers have reported patients quitting when they learned that they had a cardiovascular disease [11]. The more severe the condition, the greater the like hood of giving up smoking [11]. In fact, health concern is the most often revealed in the literature containing reasons for quitting tobacco consumption [26,28-30].

However, from a public health point of view, it would be better to encourage smokers to give up smoking before serious smoking-related health problems arise [28]. Although GATS did not assess the health status of respondents at the time of study completion, former smokers mentioned concerns about health hazard of smoking and price increase of cigarettes as the most important reasons for quitting. In the study conducted in Poland by Sieminska et al., similarly to GATS the most important reason for quitting smoking was general health concern (57%). Other reasons were personal health problems (32%) and social reasons (32%). But we cannot compare those results directly because of differences in questionnaires that were applied in both studies.

Of notable interest, the implementation of public smoking bans was not cited by participants as a reason for wanting to quit smoking. Smoking ban in public places main focus is to protect nonsmokers from exposure to environmental tobacco smoke and cessation is the second aim. Nevertheless, implementing this strategy also increases quitting among smokers [4]. On the other hand, in Poland second hand smoke policies did not work appropriately. This can be explained by the fact that in Poland, total smoking bans do not cover all public places. There are numerous exceptions including bars, restaurants and worksites where smoking rooms are allowed. Furthermore, this policy can be ineffective as a tool to increase cessation rates due to poor enforcement [31]. Another interesting finding is that while high costs of cigarettes was pointed out as a second the most important reason for smoking cessation among all former smokers, it appeared to be a weaker motivator to quit for unemployed male respondents compared to the employed (Table 2). It can happen that after price increase economically disadvantaged smokers switch from manufactured cigarettes to hand-rolled or less expensive brands or buy illegal products however not give up smoking. Nonetheless, many smokers continue the habit even in spite of smoking-associated increase of socio-economic inequalities, because disadvantaged smokers are not motivated to quit, and rather spend more money on tobacco, less to other goods what deepens deprivation. It suggests that aside fiscal policies, other policies should be used to increase cessation among lower socio-economic groups.

Our findings which show that the probability of successfully quitting smoking in employed men was markedly higher compared with unemployed men is in accord with previous evidence [8,26,32]. Positive association between success in quitting and socioeconomic resources is well established [12,33,34]. Possible explanations for the lack of the association of quitting smoking with employment among women in Poland, is the low participation of females in the labor market, and the traditional more involvement of women in household keeping, raising children and dependency on partner’s wages [21]. It appears that this indicator does not adequately reflect women’s social classes and economic positions. Similarly, association of employment with prevalence of current smoking was not found among women in Poland [21]. Moreover, we take these results with caution because one’s employment status may change over the life span, but due to cross-sectional study design we can observe only one point of the time.

Another important factor increasing quit rates is increasing levels of education. Factors that may potentially contribute to inequalities in quit rates between respondents with higher and lower levels of education may include general knowledge about health, attitudes and social norms. People with lower levels of education may have less consideration or awareness of health risks of smoking and environmental tobacco smoke [21]. GATS showed a very strong correlation between educational level and successful cessation. This correlation is consistent with other findings [3,7,20,32,35,36]. Schaap et al., in the study of effects of nationwide tobacco control policies on smoking cessation in high and less educated groups in 18 European countries, noted that smokers with higher education were more likely to have quit smoking than smokers with lower education in all age-sex groups in all countries [37]. Additionally, this study showed that countries with more developed tobacco control policies have higher quit ratios than countries with less developed tobacco control policies. Several tobacco control measures were introduced in Poland during the past decades including ban on cigarette advertising, taxation of tobacco, partial ban of smoking in public places or text of health warnings on packs of cigarettes [6,31]. However anti-tobacco policies are still not comprehensive in our country and there is a ground for further improvement including implementation of pictorial health warnings, media campaigns or 100% smoking ban in public places [31].

Another factor that we assessed in our study was potential correlation between successful cessation tobacco use and religiosity. We found that being religious did not influence cessation rates in female and male religious (but not practicing) respondents, and they were less likely to quit over a year period than the non-religious ones. Poland is mostly a Catholic country and the abstinence from substance abuse is one of the principles of the church. On the other hand, religiosity is a very sensitive issue and maybe some respondents preferred to answer that they are religious but not practicing than to state that they are not religious. We can only speculate that they decided to choose more socially acceptable answers which influenced our observation. There is no clear explanation for this finding and we do not have other nationally representative statistics to compare with the GATS results. Unfortunately, this aspect remains unexplored and again more in depth studies are needed to investigate this association in our country. Nevertheless, it is worth addressing that according to some authors religion may play a part in health beliefs and behaviors such as tobacco use [38]. Reports suggest that religiosity in different faiths is associated with less use of tobacco [38,39]. Existing studies focus on influence of many different religions; for example Kabat et al. set that Jews had significantly higher quit rates compared to non-Jews [7]. However some authors suggest that members of the same community, even if they adhere to different faiths, seem to have similar patterns of tobacco use. Maziak et al. have previously discussed that where differences occurred, it is not clear whether this is due to religion or to broader social differences of which religion is only one [40].

Although we did not find an association between age of smoking initiation and quitting in the multivariate analysis in other studies have shown that, older age of smoking uptake was correlated with increased cessation rates [10,32]. Breslau et al. reported that likelihood of smoking cessation was greater in smokers who had begun cigarette smoking after age 13 than in those who had begun earlier. Compared with smokers in the earliest initiation group, smokers who began at 14 to 16 years were 1.6 times more likely to quit, and those who began at age 17 or later were twice as likely to quit [32]. Similarly, in the study by Khuder et al., men who started smoking before 16 years of age had an odds ratio of 2.1 (95% confidence interval: 1.4–3.0) for not quitting smoking compared to those who started at a later age [41]. Higher quit rates among those who started smoking on regular basis at a later age may be due to the fact that these groups are probably less habituated and can quit more easily. Evidence shows that nicotine dependent smokers have more difficulty quitting and are therefore less likely to quit successfully than non-dependent smokers [32]. In the study of Breslau et al., smokers with nicotine dependence were 40% less likely to quit than smokers who were not dependent [32]. According to a recent study by Marquese-Valdez at al., difficulty to quit increased with increasing nicotine dependence and the number of previous quitting attempts [28]. In the population of International Tobacco Control (ITC) Four Country Study, Hyland et al. also found [10] that lower levels of nicotine dependence was the main factor that predicted future cessation among those who made a quit attempt. Existing data suggests that programs that delay smoking initiation might have significant value even if they do not lead to fully preventing the uptake of smoking [32]. It is suggested that delaying smoking initiation among adolescents could in the long run reduce the rate of smoking through increasing the potential for successful cessation [32].

It is remarkable and relevant to health policy planners and tobacco control professionals that high numbers of current smokers were not discussed with and advised to quit. Also, a very small number of those who made a quit attempt used any aid. Aveyard et al. has stressed that brief interventions – doctors advising patients to stop smoking- is the simplest approach that can increase smoking cessation [42]. This relatively easy to accomplish, non-time-consuming procedure, has been found to be effective [43]. According to Stead et al. most patients who receive such advice will not act on it, and most people who act on it will not succeed at the first attempt [43]. Regardless, this intervention is of vital importance to public health and it is important to maximize its effectiveness. The most common intervention doctors make is to advise cessation (because it will prevent ill health), but offering support for smoking cessation (such as medication or behavioral support) enhances the rate at which people attempt to stop smoking [4,42,44]. In Poland, nicotine replacement medications are available over the counter while Varenicline and Buproprion are available on prescription [6]. None of these medications are reimbursed even partly by the NHF and, therefore, many smokers who want to quit cannot afford these treatments. National Health Fund (NHF) reimburses just smoking counseling. In addition, funds assured for this activity are very limited whereas financial incentives are seen as an approach to encourage more systematic use of smoking cessation interventions by healthcare professionals [45]. Financial incentives appear to improve recording of smoking status, and increase the provision of cessation advice and referrals to stop smoking services [45]. The anti-nicotine intervention should be an essential component of the primary medical services contracted by the National Health Fund. Moreover healthcare professionals need to be adequately prepared and paid for providing relevant anti-nicotine interventions [6]. The reach of interventions needs to be broadened to increase quit rates in Poland.

### Study limitations

GATS provided nationally representative data based on a high number of respondents including current and former smokers. However the well-known potential limitation is using self-reported techniques to obtain data, but this issue was discussed in previous papers and should not substantially influence the quality of the study [21,23]. Although GATS questionnaire included questions on duration of tobacco smoking and age of smoking onset, the nicotine dependence or number of cigarettes smoked per day that are considered important determinants of cessation were missing for long-term quitters. Similarly, data on aided or unaided quitting are not available for respondents maintaining cessation for one year or longer. Moreover, the information on number of quitting attempts and their duration needed for successful cessation were omitted. Data on annual household net income as well as marital status should also be taken into consideration in future surveys because these variables may contribute to quitting [11,30]. Moreover the cross-sectional nature of this study limits the ability to draw conclusions about the directionality of the findings. A different limitation of the GATS project is that all information was collected at the time of interview [7]. While some of the characteristics may not have changed over time, others such as employment status may have changed and we cannot check past circumstances. This limits our ability to conclude on some factors like employment status as a predictor of smoking cessation.

## Conclusions

In contrast to more developed countries there is still the lack of steady tobacco surveillance system in Poland. Thus we do not have nationally representative data produced on annual basis. Literature on predictors of tobacco smoking cessation in Poland is relatively undeveloped so far. To our knowledge, GATS is the first study assessing simultaneous impact of several socio-demographic indicators on successful cessation among Poles, and probably the first to consider religiosity in such analysis. GATS provided key policy implications. In spite of some limitations of GATS we have indicated several significant associations of long-term smoking cessation including older age, high education attainment, employment in men and awareness of smoking health consequences among both genders. These findings may give framework for tailoring effective antismoking measures addressing adults. Apart from improving the effectiveness of the cessation broadening reach of such services, which is essential, cessation programs also need to address younger age groups and less likely to quit groups of population. Moreover, interventions to raise awareness on smoking health risks and quitting benefits are fundamental to increase cessation potential among adult smokers. This study clearly shows that other aspects of tobacco control in Poland like smoke-free policies should be expanded in order to increase the quitting rates. However, still crucial to preventing tobacco related diseases and maintaining good health is to never start smoking. Thus future research is also needed to examine indicators that contribute to the initiation of smoking among Poles to increase comprehensiveness of anti-tobacco policies.

## Competing interests

The authors declare that there are no conflicts of interest.

## Authors’ contributions

DK led the project, outlined the paper, discussed core ideas, and prepared the final manuscript. PK prepared the dataset, did the data analysis, TMD drafted the paper discussed core ideas and commented extensively on drafts. BU drafted the paper, and did the literature search. LBR commented on drafts. AF discussed core ideas and commented on drafts. All authors read and approved the final manuscript.

## Pre-publication history

The pre-publication history for this paper can be accessed here:

http://www.biomedcentral.com/1471-2458/12/1020/prepub
